# Philadelphia Hospital

**Published:** 1844-02-24

**Authors:** 


### PHILADELPHIA HOSPITAL.

### CLINIC OF THE JEFFERSON MEDICAL COLLEGE.

*January 27th*, 1844.

BY PROFESSOR DUNGLIGSON.

(Reported by Dr. C. J. Clarke, of Alabama.)

### CARDIAC DISEASE.

Dr. Dunglison commenced his lecture by a few remarks in reference to the case of cardiac disease brought before the class at his last clinical lecture, which presented such difficulties in diagnosis. He exhibited a drawing illustrating the post mortem appearances of a case that had occurred in the wards about a year ago, and that presented somewhat analogous symptoms: a large aneurism had formed at the arch of the aorta, and anterior to it an extensive abscess which had penetrated the walls of the thorax; and had obscured the diagnosis.

The patient whom he presented at the last lecture, remains in much the same condition, excepting that the external tumour is gradually increasing in size.

### CHRONIC GASTRITIS.

The Professor said he would bring another case before the class, which also presented difficulties in diagnosis, but of a different character from the former.

A labourer, 38 years of age, whose habits have been temperate for the last 11 years, about a year ago, for the first time, felt uneasiness in the region of the stomach, accompanied by sharp lancinating pain, acidity, sense of weight in the stomach, tumefaction of the epigastric region after eating, and occasional vomiting of a thick mucus, tinged with a fluid of a dark pitchy color; appetite unimpaired, bowels inordinately constipated, cathartics having but a temporary effect.

He was in the Pennsylvania Hospital last July, where he was treated by revellents and mild laxatives, under which he greatly improved, and at the end of a few weeks was discharged. In November last, the uneasiness in the region of the stomach, with the accompanying symptoms again returned, but he had no medical aid until he entered the Hospital. During the month (November,) he had two attacks of vomiting, of an acid, bitter, reddish mucus. He entered the Hospital on the 29th of December, complaining of these gastric phenomena, with pain in the back between the scapulæ, which he suffers from only when he has acidity of stomach. He was cupped and had blisters applied over the epigastrium, and was put upon laxatives and tonics, as infusion of gentian and rhubarb, with bicarbonate of soda, as an antacid, which greatly relieved him.

Within the previous four days he had had three attacks of vomiting, preceded by a sense of fulness in the stomach, &c.; the vomiting not being spontaneous, however, but excited by introducing his finger into the throat. He has no fever, and sleeps well at night. No solid tumour can be discovered in the region of the stomach.

The Professor has examined him on different occasions, and cannot find that any solid viscus is indurated. If there were any infarction or induration of the neighboring solid viscera it might give rise to hyperæmia of the gastric mucous membrane, and to transudation of blood. Is this a case of ordinary chronic gastritis? Is there what the French call a *vice?* Is there, in other words, a cancerous cachexy? The patient does not exhibit any characteristics of the cancerous diathesis, though his color is far from that of health. He does not vomit immediately upon eating, but after a sufficient time has elapsed for the food to be partly digested, and to be sent on towards the pylorus, which is probably the seat of the disease. This gastrorrhœa resembles, in some respects, that which occurs from the other mucous membranes—as the urethra, nasal passages, &c. As there is no marked evidence of a cancerous *vice*, it will be well to treat the disease by revellents to the epigastrium, and to administer antacids internally, keeping the bowels open, and properly regulating the diet. If we were satisfied that it was a case of cancerous cachexy, we should put him on eutrophics, or on agents that are calculated to modify the condition of the circulating fluid, and through it the nutrition of the whole system. The lecturer would be disposed to prescribe, for instance, iodine in association with conium—the former as a eutrophic; the latter to allay irritation.

[The lecturer here exhibited the matter vomited, and some of it which had been diluted with water and exposed to a temperature above 167° of Fahrenheit; this showed the dark matters to be blood, the albumen of which had coagulated.]

This case certainly presents some difficulties in diagnosis. We can determine the seat, but not with certainty the precise nature of the disease. The Professor added, that he was desirous of placing before the class such cases as this and the former that they might be prepared to meet them when they went into practice, especially as young physicians are apt to be disheartened, and to believe that difficulty in diagnosis may be referable to their want of sufficient experience, when it may be really owing to the intrinsic obscurity of the case.

### ERYSIPELAS.

A woman was next presented having *erysipelas œdematosum* of the face. Erysipelas sometimes occurs endemically in the wards of the Hospital. It frequently attacks the face—*Erysipelas faciei*—and is a functional expression of a constitutional disease. Dr. Dunglison, on this account, rarely treats the *eruption* any more than he does the eruption of measles or scarlatina: his main attention is directed to the constitutional condition. Erysipelas has appeared epidemically in connexion with, and as an *expression* of fever, in different parts of our country, and has been called, in some places, "black tongue fever." The Professor believes that epidemic fever exhibits different expressions under different circumstances. Typhoid fever is a form of adynamic fever, which is often manifested on the mucous membrane of the intestines. In other instances, adynamic fever may be attended with an erysipelatous eruption, as in the black tongue fever, &c. The erysipelas that occurs in the wards of the Hospital may be regarded as a neuropathic inflammation, and does not, generally, bear depletion well. More frequently, the sulphate of quinia, in large doses, is given with advantage. In many of the cases it is well to deplete first, and then afterwards resort to the tonic course. One local agency, which the lecturer uses, is to cover the face with cotton, so as to exclude the air. In this respect he treats it in the same manner as he would a burn. It is the admission of the air in such cases that gives rise to so much irritation, and therefore, it should be excluded. Lime water and linseed oil, when applied to burns, exert a beneficial influence mainly by filling up the areolæ of the rag, thus preventing the access of air, and partly by keeping the surface moist. They can have no healing properties. On the same principle an old fellow student of the Professor's paints over the burnt surfaces after explosions of carburetted hydrogen, in the coal mines of Newcastle, England, with a compound of resin, turpentine, and wax, leaving the varnish on until the inflammation has passed away; and this plan has been attended with great success. The woman before the class, got well of the erysipelatous fever, but this peculiarity has been left—that if a blister be applied, which she has done occasionally for severe headache, it brings on a return of the erysipelas in a local form, and of the œdematous character. It appears to be, in her case, local, or *erythema*, to be owing to a predisposition left by the former attack, and requires local treatment only: indeed the fluid is readily taken up, and the erythema disappears in a few days, without the use of any remedies.

### CONTRACTED CHEST FROM CHRONIC PLEURISY.

A man was next introduced, who had contraction of one side of his chest, caused by the absorption of the effusion from chronic pleurisy of the left side, and atrophy of the lung induced by the pressure of the fluid. The difference between the two sides was 1½ inches—one inch smaller than in health. Dr. Dunglison had suggested before, when commenting on his case, that it would probably go on to tuberculization. He also had remarked that, if the lung would remain stationary, it was the most favorable termination which could be expected. Since that time there has been a gradual return, but to a slight degree only, of the natural respiratory sounds.

This man, without taking any thing—for his condition suggested nothing—is then, by the recuperative powers of his system alone, regaining a little more use of the affected lung. The lecturer hoped that there are no tubercles forming. In such cases, care must be taken not to injure the powers of the system by drug treatment of any kind; for on those powers trust must be reposed for restoring the lung to the healthy condition, if this be practicable. During the inactivity of the diseased lung, the respiration will be carried on by its fellow. The Professor added, that mere contraction of chest, and absence of respiration in a lung, do not necessarily require treatment, and he referred to the fact, that when he was examining the chests of different persons in the wards not complaining of pulmonic symptoms, in order to deduce the average difference between the two sides of the chest in health, he was surprised to find, in one case, a difference of 1½ inch between the sides, with every physical sign of absence of respiration in the lung and the side, and yet the individual had no recollection of ever having suffered from disease of the chest. He had probably laboured under chronic pleurisy, which had terminated in atrophy of the lung—as favorable a termination, as before remarked, as under the circumstances might, perhaps, be expected.

### ANOMALOUS SENSATIONS.

There had been an amputation of the thigh performed by Dr. Pancoast, before the class, immediately before Dr. Dunglison's clinic, to which he now adverted, for the purpose of making a remark or two in elucidation of the subject of sensation, on which he had been engaged during the week in Jefferson Medical College. He said the main pain attending cutting was caused by the division of the integuments, and had been described by Dr. James Johnson, who had repeatedly felt it, as being a sensation like that which would be produced by the pouring of melted lead on the parts.

A peculiar phenomenon following amputation is a sense of itching or pain in some part of the amputated limb—as the great toe or ankle—and which the class might have the opportunity of verifying by inquiring of the patient. The lecturer referred to this point, in order to comment upon the idea entertained by some physiologists, that in all cases in which we recall pictures to our mind of objects which we have seen in dreaming, for example, the action must commence in the brain and be conveyed back along the optic nerves to the retina, so as to impress the retina, from within, as the objects originally impressed it from without. He thought the phenomena in the case of amputated limbs did not favour this view, inasmuch as the sense of itching or pain was referred to a part of a limb which did not really exist, or which had been removed from the body.

### SOFTENING OF THE BRAIN AND HEMIPLEGIA.

The attention of the class was again called to a case of *ramollissement* of the brain and hemiplegia, which had been exhibited to them before.

The Professor remarked, that when these cerebral hemorrhages had once occurred, there was great danger of another attack. Since that time, a second attack had been experienced, and a marked increase of the paralysis. When, as in this case, there is a softening of the brain, the danger of subsequent hemorrhage is much greater; and the individual generally dies, sooner or later, from the disease. He concluded by remarking that he would make this case the subject of comment at the next lecture.

